# Nrf2-mediated anti-inflammatory polarization of macrophages as therapeutic targets for osteoarthritis

**DOI:** 10.3389/fimmu.2022.967193

**Published:** 2022-08-12

**Authors:** Lin Wang, Chengqi He

**Affiliations:** ^1^ Institute of Rehabilitation Medicine, West China Hospital, Sichuan University, Chengdu, China; ^2^ Key Laboratory of Rehabilitation Medicine, West China Hospital, Sichuan University, Chengdu, China

**Keywords:** Nrf2, macrophage, osteoarthritis, intra-articular, anti-inflammation

## Abstract

Macrophages are the most abundant immune cells within the synovial joints, and also the main innate immune effector cells triggering the initial inflammatory responses in the pathological process of osteoarthritis (OA). The transition of synovial macrophages between pro-inflammatory and anti-inflammatory phenotypes can play a key role in building the intra-articular microenvironment. The pro-inflammatory cascade induced by TNF-α, IL-1β, and IL-6 is closely related to M1 macrophages, resulting in the production of pro-chondrolytic mediators. However, IL-10, IL1RA, CCL-18, IGF, and TGF are closely related to M2 macrophages, leading to the protection of cartilage and the promoted regeneration. The inhibition of NF-κB signaling pathway is central in OA treatment *via* controlling inflammatory responses in macrophages, while the nuclear factor erythroid 2-related factor 2 (Nrf2) signaling pathway appears not to attract widespread attention in the field. Nrf2 is a transcription factor encoding a large number of antioxidant enzymes. The activation of Nrf2 can have antioxidant and anti-inflammatory effects, which can also have complex crosstalk with NF-κB signaling pathway. The activation of Nrf2 can inhibit the M1 polarization and promote the M2 polarization through potential signaling transductions including TGF-β/SMAD, TLR/NF-κB, and JAK/STAT signaling pathways, with the regulation or cooperation of Notch, NLRP3, PI3K/Akt, and MAPK signaling. And the expression of heme oxygenase-1 (HO-1) and the negative regulation of Nrf2 for NF-κB can be the main mechanisms for promotion. Furthermore, the candidates of OA treatment by activating Nrf2 to promote M2 phenotype macrophages in OA are also reviewed in this work, such as itaconate and fumarate derivatives, curcumin, quercetin, melatonin, mesenchymal stem cells, and low-intensity pulsed ultrasound.

## 1 Introduction

Osteoarthritis (OA) is a highly prevalent musculoskeletal disorder characterized by pain, deformity, and functional deficits. Globally, it is a major medical and socioeconomic burden (1). Histologically, OA is characterized by cartilage degeneration, synovial lining thickening, and subchondral sclerosis (2). In pathophysiology, a low-grade, chronic inflammation predominantly leads to synovial joint deterioration as a result of an innate immune response (3). *In vivo* imaging evidence in patients with OA indicates a crucial role for activated macrophages, which is linked to its severity. Moreover, the disruption of macrophage transition may contribute to chronic and irreversible inflammatory changes in OA-affected joints (4). The activation of macrophages with heterogeneous phenotypes, which can exert pro- and anti-inflammatory effects on articular tissues during OA, has been suggested as a potential therapeutic target (5).

The macrophages within synovial joints include resident and interstitial subsets. The interstitial population of recruited monocyte-derived macrophages could exert functions of joint inflammation. While, a tight-junction-mediated shield composing of the subset of epithelial-like CX3CR1^+^ tissue-resident macrophages could restrict the inflammatory response (6, 7). OA mainly results from innate immune response induced by macrophages that are marked by CD14 and F4/80 as general surface antigens (8, 9). Furthermore, the plastic heterogeneous phenotypes of macrophages include classically activated pro-inflammatory phenotypes (CD80, CD86, and CD11b as M1 surface markers) and alternatively activated anti-inflammatory phenotypes (CD163, and CD206 as M2 surface markers) (10–12). There are several subtypes of M2 macrophages, including M2a marked by CD206, M2b marked by CD86, M2c marked by CD163 (13, 14), and M2d marked by CD68 (15–17). Induced by IL-4 and IL-13, M2a plays a vital role in anti-inflammation response and wound-healing *via* up-regulating the expression of IL-1RA, IL-10, CCL-18, and TGF-β. M2b is the immune regulator between M1 and M2a, which serves in both pro-and anti-inflammation responses. Besides, IL-10, TGF-β, or glucocorticoids can induce M2c activation, which causes the secretion of IL-10, CCL-18, and TGF-β, and M2c also has a powerful phagocytosis function (18, 19). While, M2d, also called tumor-associated macrophages (TAMs), plays a vital role in potent immunosuppression, angiogenesis, wound healing, and cancer metastasis (15, 16). However, most experimental therapies or mechanisms for OA use the balance between M1 and M2 as a key point (5, 20–22). In this review, the polarization state of macrophages will be dichotomized without discussing specific subtypes of M2. In addition, synovia in OA patients has a substantial proportion of M1 macrophages, which is consistent with CD14 and CD163 expression levels (23). Macrophage phenotypes are influenced by intracellular redox metabolism, as evidenced by increasing studies, leading to metabolic reprogramming from glycolysis in M1 to oxidative phosphorylation (OxPhos) in M2 (5, 24–26).

Nuclear factor erythroid 2-related factor 2 (Nrf2) is a transcription factor expressed in most tissues and cells at a low level in the cytoplasm under homeostatic conditions through binding to Kelch-like ECH-associated protein 1 (KEAP1). Nrf2 is released in response to stress signals sensed by KEAP1, translocates to the nucleus, accumulates, and binds to antioxidant response elements (AREs) of target gene promoters (27). Finally, heme oxygenase-1 (HO-1), and glutathione S-transferase (GST) are transcribed, and reactive oxygen species (ROS) removal systems are initiated to protect cells from oxidative stress-induced damages and maintain redox homeostasis (28). Besides, there is increasing evidence that quite different metabolic characteristics and inflammation phenotypes between M1 and M2 macrophages are highly dependent on Nrf2. The negative regulation of Nrf2-related signalings for other transcription factors, such as nuclear factor-κB (NF-κB), may shed light on the link between defense against oxidative stress and reducing inflammation through Nrf2 signaling (24, 29). For example, cyclooxygenase-2 (COX-2), and hypoxia-inducible factor-1α (HIF-1α), which are closely related to the M1 phenotype, could be suppressed *via* the activation of Nrf2 signaling (30). Moreover, the activation of Nrf2 in macrophage could increase the levels of cysteine and glutathione (GSH), by regulating the transporter between cysteine and glutamate, and the GSH-synthesizing enzyme (31, 32). GSH could suppress ROS as a major cellular antioxidant *via* activating HO-1. It has also been reported that the accumulation of GSH could induce an increase in inflammatory factors including NO, IL-1β, IL-4, IL-10, TNF-α, and PGE2, which are related to the M1 phenotype (33). However, in tubular injury resulting from oxidative stress and inflammatory response, the deletion of ROS could promote M2 polarization (34). Similarly, inflammatory response and oxidative stress could be reduced in the cardiac injury induced by LPS, during which process the Nrf2/HO-1 pathway is activated, and the GSH is accumulated (35). Furthermore, it has been reported that the activated Nrf2 could suppress IL-1β without NF-κB or GSH in alveolar macrophages (36). In summary, the relationship is complicated, between the macrophage polarization and metabolic adaptation of macrophages upon inflammatory response and oxidative stress, including OA. As potential mechanisms underlying the protective effects of Nrf2 activation in macrophages, the Nrf2/HO-1 signaling pathway, and the negative regulation of NF-κB signaling will be discussed later.

Furthermore, it has been shown that there is extensive crosstalk between transcriptional pathways involving Nrf2 and NF-κB during oxidative stress and inflammation (29, 32). Generally, Nrf2 signaling negatively regulates NF-κB signaling in oxidative stress and inflammatory response, especially NF-κB (P65) pathway (37). That is, through Nrf2 transcriptional activation, the redox status and metabolism of macrophages changes, resulting in an anti-inflammatory phenotype (38). Stimulation inducing inflammatory M1 can simultaneously initiate NF-κB-dependent transcriptional pathway inducing the secretion of inflammatory factors quickly, and initiate Nrf2-dependent transcriptional pathway at the same time to cytoprotective response slowly (39). In summary, the action of Nrf2 has a potential role in preventing M1 polarization, and then promoting chondral protection and inhibiting OA progress. For example, in OA, Ca^2+^ influx evoked by transient receptor potential vanilloid 1 (TRPV1) mediated inhibition of M1 macrophage polarization through the phosphorylation of calmodulin-dependent protein kinase II (CaMKII), while the specific inhibitor of Nrf2 counteracted the anti-inflammatory effect (40).

However, the role of Nrf2 in macrophage reprogramming for OA treatment is still unclear to a large extent. Therefore, this review synthesizes evidence of macrophage reprogramming induced Nrf2 inhibition or activation in the progression or treatment of OA.

## 2 Osteoarthritis pathology driven by macrophages

### 2.1 M1-induced intra-articular inflammation

The intra-articular microenvironment is characterized by an inflammatory infiltrate largely composed of synovial macrophages. Inflammatory macrophages are believed to be responsible for the presence of OA (23). Damage-associated molecular patterns (DAMPs) are molecules or fragments produced by initial harmful factors that can trigger innate immunity. The DAMPs could activate pattern-recognition receptors for macrophage activation, for example, the toll-like receptor (TLR) 4, and the ligands could be cartilage matrix fragments, or plasma proteins into the articular cavity in OA (41, 42). In contrast, the production of macrophage-derived pro-inflammatory cytokines, such as TNF-α and IL-1β, was greatly reduced by depleting CD14-positive synovial macrophages specifically from OA synovial cells (43).

M1 macrophages tend to secrete pro-inflammatory cytokines including TNF-α and L-1β (44). A series of events triggered by TNF-α and IL-1β can cause cartilage degeneration, where chondrocyte death and cartilage matrix degradation are accelerated while synthesis and regeneration are inhibited (45). Cartilage mainly consists of chondrocytes and extracellular matrix (ECM). Apoptosis of chondrocytes and degeneration of aggrecan (ACAN) and type II collagen (COL2) in ECM are prominent pathological changes in OA (46). It is believed that autocrine TNF-α and IL-1β trigger the pro-inflammatory events in chondrocytes and the catabolic cascades in fibroblast synoviocytes through NF-κB signaling, resulting in the production of IL-6, NO, and prostaglandin E2 (PGE2) (3, 47). The release of IL-6 from macrophages induced by IL-1β can stimulate STAT3 signaling in macrophages, enhancing inflammation responses (48). As a result of IL-6 stimulation, chondrocytes and synovial fibroblasts produce PGE2 and collagenase (49). The high level of NO can inhibit the synthesis of ECM and enhance the activity of matrix metalloproteases (MMPs) (45). By degrading collagen and digesting matrix proteins, MMP1, 3 and 13 can result in skeletal cartilage absorption. The metabolic product of activated COX is arachidonic acid, the substrate of PGE2 biosynthesis (50). PGE2 can also stimulate the release of IL-6 through activating NF-κB pathways (49).

The increase of expression of one of the specific receptor of TNF-α, called TNF receptor I (TNFRI or p55), has been found on OA chondrocytes and synovial fibroblasts (47). In fact, TNF-α plays a central role in the intra-inflammation cascades of OA. TNF-α can break down the cartilage by inhibiting the synthesis of proteoglycan and COL2, and also promoting the apoptosis of chondrocytes. The death domain (DD) in the TNF receptor superfamily is a cytosolic domain and a cysteine-rich extracellular domain. The extrinsic apoptosis pathway is governed by TNF-α, which binds to and interacts with DD, acting on downstream caspases, ultimately leading to apoptosis (45). Similarly, the expression of the specific receptors of IL-1β, called IL-1 receptor type I (IL-1RI), has been found to be increased in human chondrocytes and synovial fibroblasts affected by OA (47). Furthermore, NO can decrease the level of IL-1RA (the antagonist of IL-1R) leading to an increase in IL-1 production (51). IL-1β can also induce apoptosis of chondrocytes relying on endogenous NO in reverse (45). In synovial fibroblasts, IL-1β activates NF-κB (P65) and promotes transcription of IL-6 and PGE2 (52). In chondrocytes, IL-1β can up-regulate the expression of IL-6 through phosphorylating STAT1 and STAT3 (45).

MMPs are a superfamily of proteases that can remodel and degrade ECM in connective tissues. By stimulating the release of MMP1, MMP3, and MMP13 in chondrocytes, TNF-α and IL-1β can affect the synthesis of proteoglycans, connexins, and type II collagen (47). MMP1 and MMP13 are collagenases, while MMP3 is matrix lyases. Apart from that, ADAMTS-4 and ADAMTS-5 belong to the disintegrin and metalloproteinase with thrombospondin motifs family (ADAMTS), which destroys ECM independently from MMPs (53). In chondrocytes, TNF-α and IL-1β can induce an increase in the release of ADAMTS-4 and ADAMTS-5 (45, 54). In addition, direct evidence shows the M1 inflammatory secretion due to interferon-γand TNF-α inhibits chondrogenic differentiation and cartilage repair by up-regulating IL-1β, IL-6, NO, MMP13, and ADAMTS5, and down-regulating ACAN and COL2 (55). To sum up, DAMPs-induced activation of M1 macrophages mainly dependent on NF-κB signaling mediates intra-articular inflammation and cartilage degeneration in OA.

### 2.2 M2-induced intra-articular anti-inflammation response

The failure in the appropriate proportion of M1 and M2 phenotypes can be the main cause of OA-related low-grade inflammation (56). After all, M2 macrophages are anti-inflammatory and help to repair cartilage in contrast to M1 macrophages. In particular, M2 macrophages can secrete anti-inflammatory factors including IL-10, IL-1RA, chemokine (CeC motif) ligand (CCL18), and pro-chondrogenic mediators including transforming growth factor β (TGF-β) and insulin-like growth factor (IGF) (57). M2 phenotype can be activated by IL-4, IL-10, IL-13, TGF-β, and CCL18, which lead to a positive feedback loop to resolute inflammation (58, 59). Besides, M2 can also regulate collagen turnover pathways in cartilage to promote collagen remodeling (60). The secretion of M2 type can promote cartilage repair by up-regulating COL2 and glycosaminoglycan, inhibiting MMP13, and inhibiting apoptosis of chondrocytes (57).

The previous evidence indicates that IL-10 can protect and repair cartilage, and contributes to the regenerative microenvironment. In patients with OA, IL-10 reduces the specific receptors for TNF-α, and the effects of TNF-α on the fibroblasts by down-regulating PEG2, COX-2, and PLA2 (61). By modulating mitochondrial apoptotic pathways, IL-10 can also inhibit chondrocyte apoptosis by reducing caspase activity and the Bax/Bcl-2 ratio (62). Besides, IL-10 can promote the repairing of chondrocytes and ECM. After IL-10 is administered to compressed articular cartilage *in vitro*, the cell death of chondrocytes, the release of glycosaminoglycans, NO, and molecules that promote ECM degradation and inhibit its syntheses, such as MMP3, MMP13, ADAMTS-4, and inducible nitric oxide synthase (iNOS) are significantly reduced. Moreover, the subsequent study confirmed that the ECM protective effects of IL-10 can be time-dependent (63). Moreover, the overexpression of IL-10 can antagonize the characteristics of cartilage catabolism (MMP3 and MMP13) and the down-regulation of COL2 gene expression induced by TNF-α (64). In addition, the conditioned medium of M1 macrophages decreased the expression of COL2 and ACAN genes in mesenchymal stem cells (MSCs), which are genes associated with chondrogenic differentiation, while M2 macrophages did not exhibit similar inhibition (65).

Furthermore, the increase in IL-10 and IL-1RA can be induced by IL-4, causing the M2 activation in macrophages. The increased IL-10 and IL-1RA, as an anti-inflammatory response, also contribute to responding to the transcription of TNF-α induced by IFN-γ in macrophages. However, in OA synovial fluid, the expression of both is inhibited (66). IL-1RA can be produced by chondrocytes, monocytes, and fibroblasts, belonging to the IL-1 family, and competes with IL-1β to combine with IL-1R type I and II, without triggering the IL-1β-relative downstream inflammatory responses. Although, the production of endogenous IL-1RA needs to be 10-1000 times that of IL-1β to effectively block the binding of IL-1β (67). Additionally, CCL18 is a T-cell chemokine subset associated with the Th2 adaptive responses to IL-4, IL-10, and IL-13. CCL18 has since been identified as a mediator secreted by M2 and an introducer of M2 type (68). Besides, CCL18 can also stimulate fibroblast proliferation and collagen production independent of TGF-β (69). The role of CCL18 in OA is not extremely beneficial, because it has been seen that the relative higher CCL18 levels in synovial fluid of knee OA patients with more serious pathological structural changes (70). Besides, CCL18 can induce the significant enhancement of MMP-3 in fibroblast-like synoviocytes (71).

IGF-1 is a small polypeptide (~7 kDa) belonging to the growth factor family, of which the structure is related to insulin by 50% sequence homology (72, 73). IGF-1 can inhibit chondrocyte apoptosis induced by IL- 1β *in vitro* and can reduce synovitis in OA models (74). Furthermore, IGF-1 inhibits the degradation of cartilage ECM by down-regulating MMP-1, MMP-3, IL-1, and TNF-α (75). Notably, IGF-1 can also play a key role in cartilage anabolism by promoting COL2 and ECM proteoglycan synthesis and decreasing MMP13 to protect the cartilage (76). Through NF-κB signaling, IGF-1 can inhibit the pro-catabolism effects of IL-1β on cartilage, inhibit the apoptosis of chondrocytes (marked by caspase-3), and suppress inflammation (77–79). Moreover, it has been observed that IGF-1 promotes chondrogenic differentiation of adipose-derived MSCs through the expression of COL2, ACAN, and SOX9 (80). IGF-1 can positively regulate chondrogenesis in bone marrow-derived MSCs, and the chondroinductive effects of IGF-1 are independent of TGF-β1 (81). Furthermore, the combination of TGF and IGF can promote chondrogenesis in fracture models *in vivo* (82).

TGF-β family of growth factors and cytokines plays a critical role in skeletogenesis, and can be divided into two major subfamilies, the TGF-β/Activin/Nodal family and the bone morphogenetic protein (BMP) family (83, 84). Signaling by TGF-β1, 2, and 3 is mediated by membrane-bound receptor complexes, which are activated by SMAD proteins intracellularly. Activated type I receptor (also known as ALK5) phosphorylates SMAD2 and SMAD3, which can regulate the expression of target genes related to cartilage anabolism, COL2 for example. However, when TGF-β signals through the ALK1 receptor, phosphorylated SMADs 1/5/8 can be targeted to up-regulate cartilage catabolism genes, like MMP13 (85). The role of TGF-β in OA seems to have two sides, and the administration of TGF-β is equally controversial because the choice between phosphorylation of SMAD2/3 or SMAD5/8 has an unknown mechanism (86).

**Figure 1:**
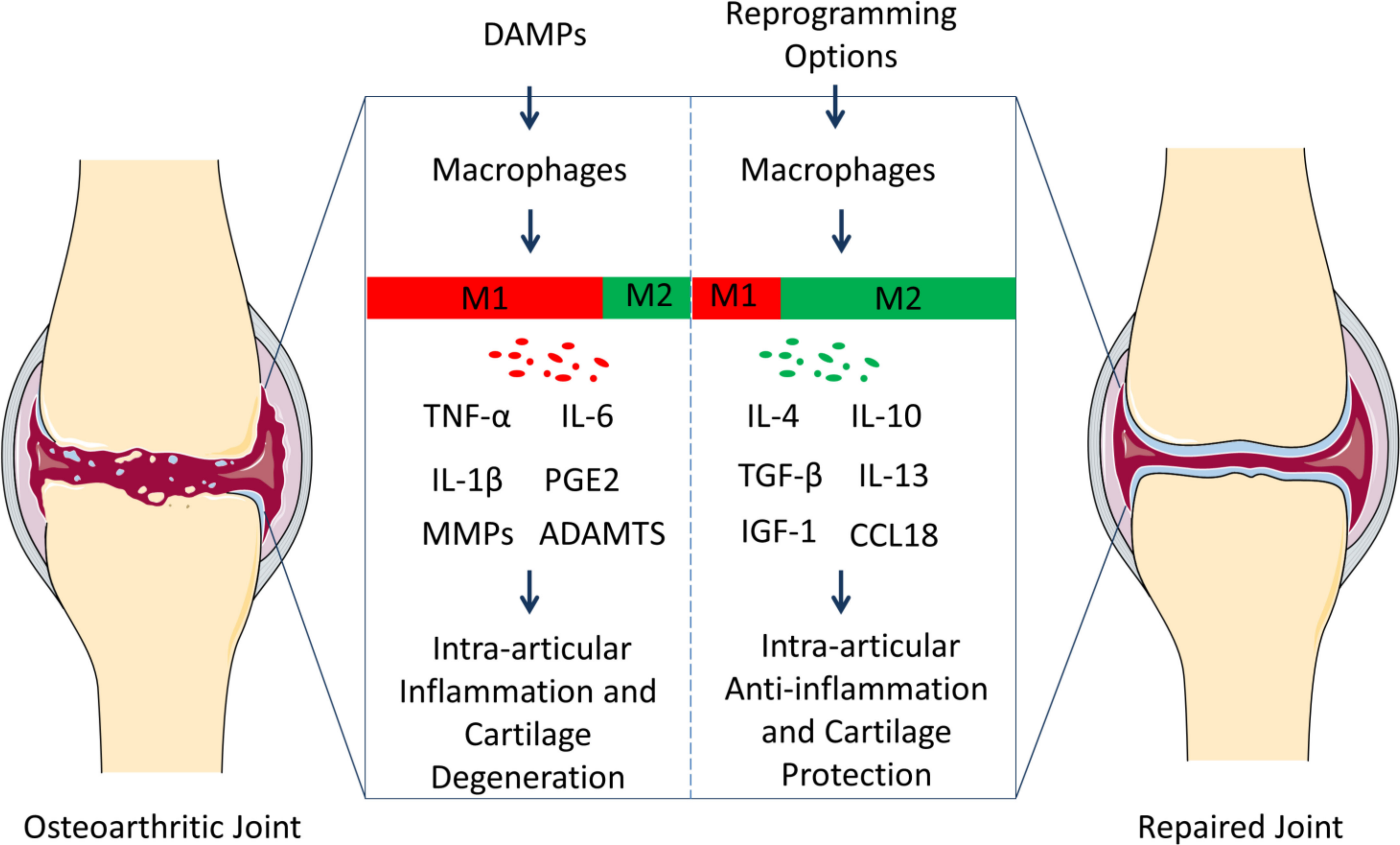
Polarization of macrophages in knee osteoarthritis (OA) pathology and repair. Resident macrophages in the synovium have two general phenotypes, M1 and M2. Macrophages tend to serve as M1 phenotype under the inflammation responses caused by damage-associated molecular patterns (DAMPs). M1 subgroups can contribute to OA progression through releasing inflammatory and degenerative molecules including TNF-α, IL-6, IL-1β, PGE2, MMPs, ADAMTS, which leads to synovitis, the death of chondrocytes, and the degradation of extracellular matrix (ECM). However, macrophages can be reprogrammed, and the M2 population can contribute to OA treatment *via* up-regulating anti-inflammatory and regenerative molecules (IL-4, IL-10, TGF-β, IL-13, IGF-1, and CCL18). And the microenvironment can be constructed, where the tissues in the osteoarthritic joints can repair. We suggest that reprograming macrophage phenotypes can be therapeutic targets for the prevention and treatment of OA.

The conversion of the phenotype of macrophages in OA could play a role in treatment because the secreted cytokines regulate inflammation and cartilage metabolism (**Figure 1**).

## 3 Signaling pathways for reprogramming macrophages

**Figure 2:**
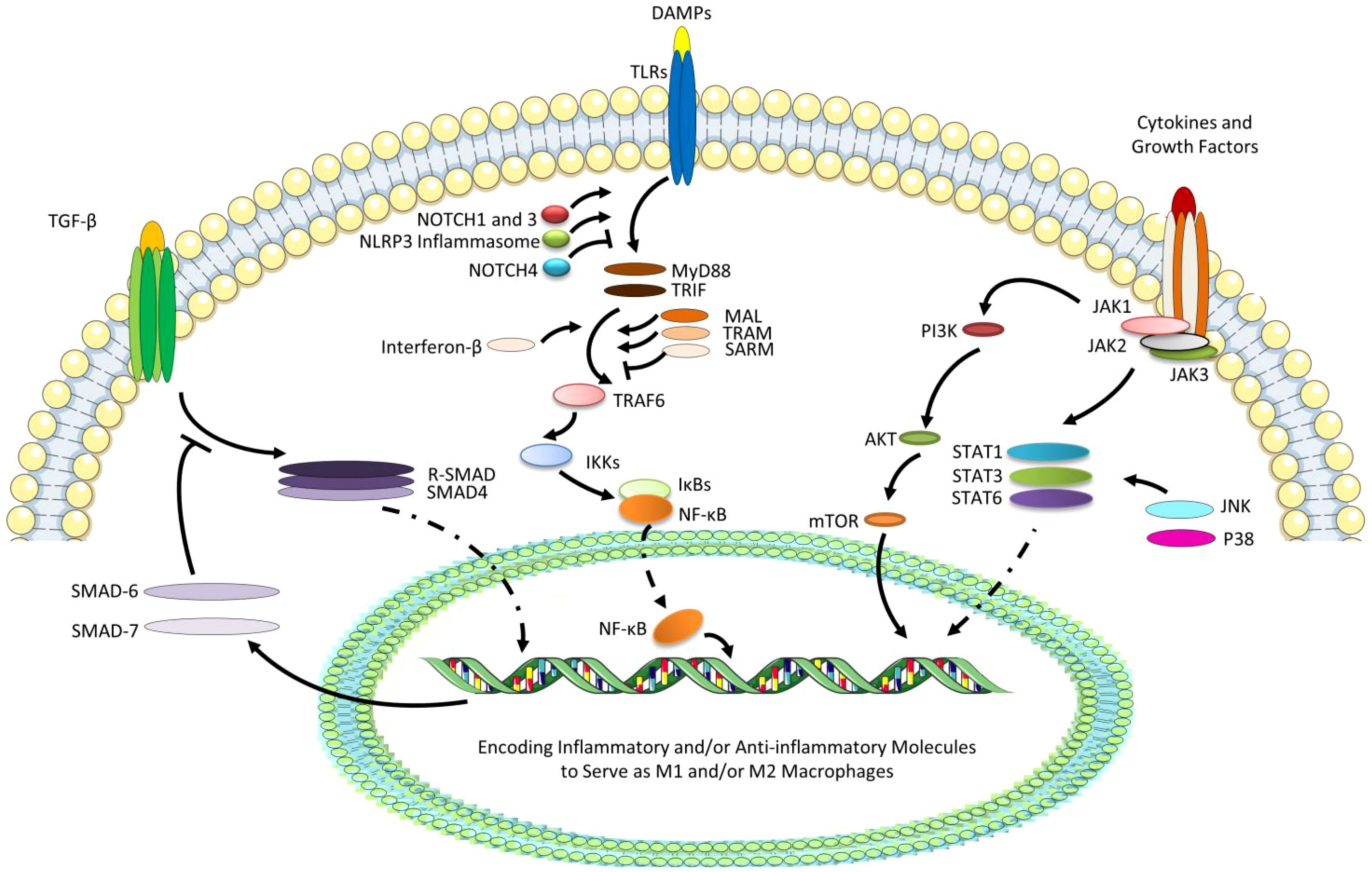
The most studied several pathways relative to reprogramming of macrophages. TGF-βcan interact with phosphorylated type II and type I receptors activating receptor-regulated SMADs (R-SMADs, a heterodimer of SMAD2 and SMAD3) to form the heteromeric trimer with SMAD4. The nuclear translocation of R-SMAD can promote the M2 polarization, while SMAD6 and SMAD7 as the negative regulators of TGF-β signaling transduction are also the target genes. Besides, due to the ligation of DAMPs with TLRs, especially TLR4, MyD88 and TRIF, TLR adaptors containing a Toll/IL-1 receptor (TIR) domain, can bind directly to TLRs and recruit MAL and TRAM, while SARM negatively can regulate the pathways. TIR-domain-containing adaptor-inducing interferon-β (dependent on TRIF) can be recruited and then activates TRAF6, and IκBs (especially IκBα) can degrade leading to the release of NF-κB (P50/65) and its translocation to the nucleus, which can promote the M1 polarization. TLR signaling cascade can also activate NOTCH and NLRP3 signaling, which can regulate pro-inflammatory responses *via* the regulated transcription of NF-κB. However, NOTCH4 can negatively regulate the TLR/NF-κB signaling. Finally, STAT/JAK signaling pathway start from tyrosine kinase-associated receptors binding various cytokines and growth factors, followed by the phosphorylation of these receptors and JAKs, and the initiation of the phosphorylation and activation of STATs. Generally, the activation of STAT6 signaling can promote M2 phenotype, while STAT1 and STAT3 can perform complicatedly under the effects of various cytokines or growth factors. JAKs can also activate PI3K/AKT signaling pathway, promoting M2 polarization, which can play a collaborative role in JAK/STAT6 signaling pathway. In addition, STATs can also be activated by JNK and P38, which belongs to extracellular signal-regulated kinases (ERKs), promoting M1 polarization.

The polarization of macrophages dynamically adapts to changes in the microenvironment, and macrophage reprogramming has a complex mechanism. The most studied several pathways relative to reprogramming of macrophages include TGF-β/SMAD, TLR/NF-κB, and JAK/STAT signaling pathways, with the regulation or cooperation of Notch, NLRP3, PI3K/Akt, and MAPK signaling (**Figure 2**).

### 3.1 TGF-β/SMAD signaling

Collagen, fibronectins, and fibrinoproteins can form complexes with TGF-β family proteins in the ECM (87). Under the effects of MMPs and/or serine proteases (for example, cathepsins), TGF-β proteins are released from the complex of ECM (86). TGF-β intracellular signaling is mediated by SMAD family members after interaction with two membrane receptors (sequentially phosphorylated type II and type I receptors) (88, 89). Receptor-regulated SMADs (R-SMADs) consisting of SMAD2 and SMAD3 are activated *via* the phosphorylated type I receptor. As a result of the heteromeric trimer with SMAD4, activated R-SMADs can translocate to the nucleus and trigger transcription of target genes (90), including more than 100 transcriptional/signaling regulators, immune modulators, and atherosclerosis-related genes. Dexamethasone-induced M2 polarization is enhanced by TGF-β/SMAD signaling, as type II receptors are elevated, which increases TGF-β response in macrophages. Several genes are associated with the M2 phenotype, including ID3, RGS1, ALOX5AP, TREM1, IL-17RB, JUNB, ELK3, RUNX3, ELL2, TLE3, BCOR, and FOS, and these genes encode functional molecules that are involved in immune responses, inhibiting apoptosis, and maintaining terminal differentiation (91).

In addition, the negative regulators of signal transduction (such as SMAD6 and SMAD7) are also the target genes of TGF-β, which regulates cell homeostasis (92). Of note, A number of E3 ubiquitin ligases, for example, the SMAD ubiquitin regulatory factors and the deubiquitinating enzymes, play a crucial role in recognition and degradation of R-SMADs, SMAD6, SMAD7, and TGF-β receptors. E3 ubiquitin ligases can induce proteasomal degradation *via* the catalysis of their substrates and self-ubiquitination (93, 94). In contrast, deubiquitinating enzymes can antagonize the ubiquitination of E3 ubiquitin ligases, for example USP15 (95). The TGF-β pathway has been studied in OA since it has the potential to regulate cartilage anabolism (86).

### 3.2 TLR/NF-κB signaling

TLRs are prototype pattern-recognition receptors (PRRs) that recognize pathogen-associated molecular patterns (PAMPs) from microorganisms or DAMPs from damaged tissue (96). TLRs are highly expressed on immune cells, including monocytes, macrophages, and dendritic cells, and can also be up-regulated in response to IL-1 or TLR-4 stimulation in other cells. Through ligation of TLRs, endogenous molecules produced during OA, such as glycoprotein, fibronectin, and hyaluronan of ECM components, have been implicated in activating immune responses (97). Also, plasma proteins can be recognized as DAMPs, such as fibrinogen, which signals through TLR4 to induce inflammatory cytokines (42). There is strong evidence that synovial macrophages and chondrocytes express TLR2 and TLR4, while TLR4 senses more DAMPs than TLR2 in both OA and rheumatoid arthritis (RA). TLR4 plays a key role in the DAMP recognition and the signaling promotion, in the forms of homodimerization or heterodimerization (for example TLR4-TLR6 heterodimers), and the co-receptors, or accessory molecules (such as CD14 and CD36) (41).

There are five TLR adaptors containing a Toll/IL-1 receptor (TIR) domain, among which MyD88 and TRIF bind directly to TLRs and recruit MAL and TRAM, respectively, while SARM negatively regulates these pathways. When the MyD88-dependent TLR4 signaling pathway mediated by TRAM is activated, TIR-domain-containing adaptor-inducing interferon-β (dependent on TRIF) can be recruited and then activates a cascade of proteins including TRAF6, which finally induces the degradation of IκBs, and the release of NF-κB (P50/65) and its translocation to the nucleus (98). NF-κB is central to all macrophage TLR-medicated inflammation responses. IκBs inhibit NF-κB in the cytoplasm by forming complexes with NF-κB. Of note, the regulation of TLR/NF-κB signaling is mainly induced by the ubiquitination/deubiquitination of TRAF6 (99). Macrophages tend to show M1 polarization secreting TNF-α, IL-1β, IL-8, and COX-2. It has been seen that the block of TLR4/NF-κB signaling pathway can inhibit M1 polarization (100–102).

TLR signaling cascade can also activate NOTCH signaling, which regulates pro-inflammatory responses *via* the regulated transcription of NF-κB (103). The Notch gene family encodes evolutionarily highly conserved, single-pass, type I transmembrane heterodimers of 300 kDa called NOTCH receptors (NOTCH1-4 in mammals) which control macrophage activation and polarization *via* TLRs (104). It has been shown that macrophages up-regulate NOTCH1 upon the activation of TLRs, which implies that NOTCH1 mainly contributes to pro-inflammatory activation (105). In particular, signaling transduction enhances transcriptional activity by phosphorylating and degrading IkBα (106). Similarly, the activation of NOTCH3 by delta-like 4 (Dll4) in macrophages leads to enhanced responses to LPS or IL-1β *via* TLR4/NF-κB pathway (107). Furthermore, NOTCH1 and NOTCH3 cooperate to control the expression of NF-κB-dependent pro-inflammatory genes following TLR-4 activation, with NOTCH3 dominating for the first hours and NOTCH1 later (104). By contrast, the inhibition of NOTCH1 can induce a decrease in M1 polarization and an increase in M2 polarization (108). However, the activation of TLRs/NF-κB pathway in macrophages is negatively regulated by NOTCH4, depending on the phosphorylation of STAT3 and the weakness of STAT1, which activates STAT3/JAK2 signaling (109). Taken together, the regulation of Notch signaling has roles with two sides in TLR/NF-κB pathway in macrophages.

Furthermore, the NLRP3 inflammasome can be activated by DAMPs/TLRs/NF-κB pathway. NLRP3 consists of microparticles, ATP, cholesterol, and microbial toxins, acting as a key sensor of tissue damage and activating sterile inflammation (110). NLRP3 interacts with adapter apoptosis-associated speck-like protein (ASC), and pro-caspase-1 is recruited as an effector, resulting in the formation of NLRP3 inflammasome in the cytosol (111). Different from NOTCH, the role of NLRP3 signaling seems to be pro-inflammation as its effects on processing interleukin precursors (such as pro-IL-1β and pro-IL-18) into mature and secreted interleukin forms (112).

Although the TLR4/NF-κB pathway in chondrocytes has been emphasized as it induces cartilage catabolism (41, 113), the signaling pathway in macrophages has also been targeted. Specifically, chondroitin sulphate inhibits NF-κB and IL-1β secretion from macrophages through inhibition of TLR4 and DAMP interactions (114). On the contrary, lumican, a glycoprotein in adult articular cartilage, has been shown to be up-regulated in OA, and induce the inflammation related to macrophages *via* TLR4 pathway (115).

### 3.3 JAK/STAT signaling

Signal transducer and activator of transcriptions (STATs) including STAT1, STAT2, STAT3, STAT4, STAT5A, STAT5B and STAT6 are latent in cytoplasm, and can be phosphorylated and activated *via* tyrosine phosphorylation and dimerization, finally leading to translocation to the nucleus, binding to promoter sequences and the activation of transcription. The transformation of STATs from latent to active is dependent on Janus kinases (JAKs), which belong to the family of tyrosine kinases (TYKs). JAK1, JAK2, JAK3, and TYK2 have been indicated as JAKs, which can act as tyrosine kinase and bind tyrosine kinase-associated receptors intracellularly. Tyrosine kinase-associated receptors can bind various cytokines and growth factors, followed by the phosphorylation of these receptors and JAKs, and the initiation of the phosphorylation and activation of STATs (116–118).

In macrophages, IL-13 and IL-4 can activate the M2 phenotype by activating JAK2/STAT3 signaling, where IL-4 phosphorylates STAT3 and STAT6, as well as up-regulating DNA binding activity of STAT3, and IL-13 initiates Tyk2 to cascade STAT1 and STAT6, and also to increase DNA binding activity of STAT1 (119). Moreover, STAT6 effects have been widely elucidated in M2 polarization induced by IL-4 and IL-13 (120, 121). IL-10 can activate STAT3 *via* JAK1 and Tyk2 (122). However, LPS and IL-6 can activate the M1 phenotype through JAK2/STAT3 signaling (123, 124). Besides, IFN-γ has an important role in phosphorylation and dimerization of STAT1, leading to M1 phenotype of macrophages (122, 125). Furthermore, STAT1 and STAT3 play antagonistic roles in pro- and anti-inflammation response in macrophages (126), whereas they can also cross-regulate each other in some immune responses, like the additional activation of STAT3 along with the pro-inflammation activation of STAT1 induced by IFN-γ (127).

Besides STATs, JAKs activate the phosphatidylinositol 3-kinase (PI3K) signaling pathway, for example, GM-CSF-induced activation of JAK2 (128). PI3Ks are lipid-signaling kinases that phosphorylate phosphoinositides to form PIP3, 4, and 5 (129, 130). After PI3K activation, 3-phosphoinositide-dependent kinase (PDK1) is recruited and activated. PDK1 phosphorylates and activates protein kinase B (AKT) (131). PI3K activation inhibits macrophage programming into M1, while AKT activation is a critical condition for M2 polarization (132). LPS induces the M1 phenotype in AKT1 ablation, while LPS induces the M2 phenotype in Akt2 ablation (133). The mechanistic target of rapamycin (mTOR) is an evolutionarily conserved PI3K family member, and contributes to the core of the downstream target signaling complexes of PI3K/AKT pathway, called mTORC1 and mTORC2 (130). In LPS-activated M1 polarization, activation of mTORC1 has been demonstrated. Furthermore, mTORC1-mediated feedback inhibition of mTORC2 activity in Akt signaling leads to inhibition of M2 polarization (134, 135). In addition, specific destruction of PI3K/AKT signaling pathway has little effect on JAK/STAT6 signaling, indicating a collaborative role for PI3K/AKT signaling pathway (136).

STATs can also be activated by serine threonine kinases other than JAKs, such as extracellular signal-regulated kinase (ERK) (137). ERK belongs to mitogen-activated protein kinase (MAPK) modules, which also include c-Jun N-terminal kinase (JNK) and P38 (also known as stress-activated protein kinases) (138), and mediate the protein kinase cascades (139). MAPK activation results in nuclear translocation of a number of transcription factors, including activator protein-1 (AP-1), activating transcription factor (ATF)-2, cAMP-responsive element binding protein (CBP), and members of the ETS family (140). In macrophages, the activation of TLR4 is induced by the phosphorylation cascade of JNK, P38, and ERK, leading to the transcription of NF-κB and AP-1, the increase in the expression of TNF-α and IL-6, forming the activation of M1 polarization (141–143). Among these, pro-inflammatory effects are mainly due to the signaling mediated by JNK and p38 (139). However, the activation of ERK1/2 can inhibit the nuclear translocation of p65 subunit of NF-κB, which results in anti-inflammation response (144, 145). Likewise, the activation of CBP/P300 can mediate the serine phosphorylation of STAT6 induced by IL-4, which can give rise to the M2 phenotype (146, 147).

## 4 Potential pathways for activating Nrf2 of macrophages in OA as therapeutic choices

Nrf2 belongs to the Cap’n’collar basic leucine zipper transcription factor family, within which the 605 amino acids act their roles as seven highly conserved functional NRF2-ECH homology (Neh) domains (148). It is the Neh2 domain in Nrf2 closest to the N-terminal that binds KEAP1 and is responsible for stabilizing the cytoplasm and degenerating it through ubiquitination. KEAP1 inhibits Nrf2, resulting in stable Nrf2 localization in the cytosol. A homodimer of KEAP1 can also be a stress sensor, recruiting and adapting the E3 ubiquitin ligase cullin-3 (CUL3). In turn, CUL3 can polyubiquitinate Neh2 lysine residues, finally resulting in degradation by ubiquitin proteasomes (149, 150).

Next, from the N-terminal to the C-terminal, there are Neh4, Neh5, Neh7, Neh6, Neh1, and Neh3, respectively. Neh4, Neh5, and Neh3 are transactivation domains that mediate the interaction of Nrf2 with other coactivators. Neh4 and Neh5 can bind CBP/P300 (151, 152), while Neh3 binds chromodomain-helicase-DNA binding 6 (CHD6), contributing to transcription. However, Neh7 and Neh6 are negative regulatory domains for Nrf2, which can bind a β-transducin repeat-containing protein (β-TrCP) and retinoic X receptor α (RXRα), separately (153, 154). The DNA binding domain of Neh1 is mediated by heterodimerization with transcription factors, such as small musculoaponeurotic fibrosarcoma (sMAF) (155).

In cells exposed to stress or electrophilic agents, the alignment of Nrf2 lysine residues is disrupted by the specific thiol residues, leading to the lysine residues being modified by electrophiles within the weak interaction with KEAP1, preventing ubiquitination, and ultimately releasing Nrf2 (156, 157). Nrf2 can translocate to the nucleus after dissociating from KEAP1. With the accumulation of Nrf2 in the nucleus, it forms a heterodimer with sMAF, which binds ARE, a trans-acting DNA enhancer motif. The Nrf2 binding ARE promotes genes encoding cytoprotective proteins, such as GSH-related enzymes, NAD(P)H dehydrogenase quinone 1 (NQO1), and HO-1, to prevent oxidative stress, electrophilic toxicity, and inflammation, and to maintain mitochondrial function and metabolism (158).

**Figure 3:**
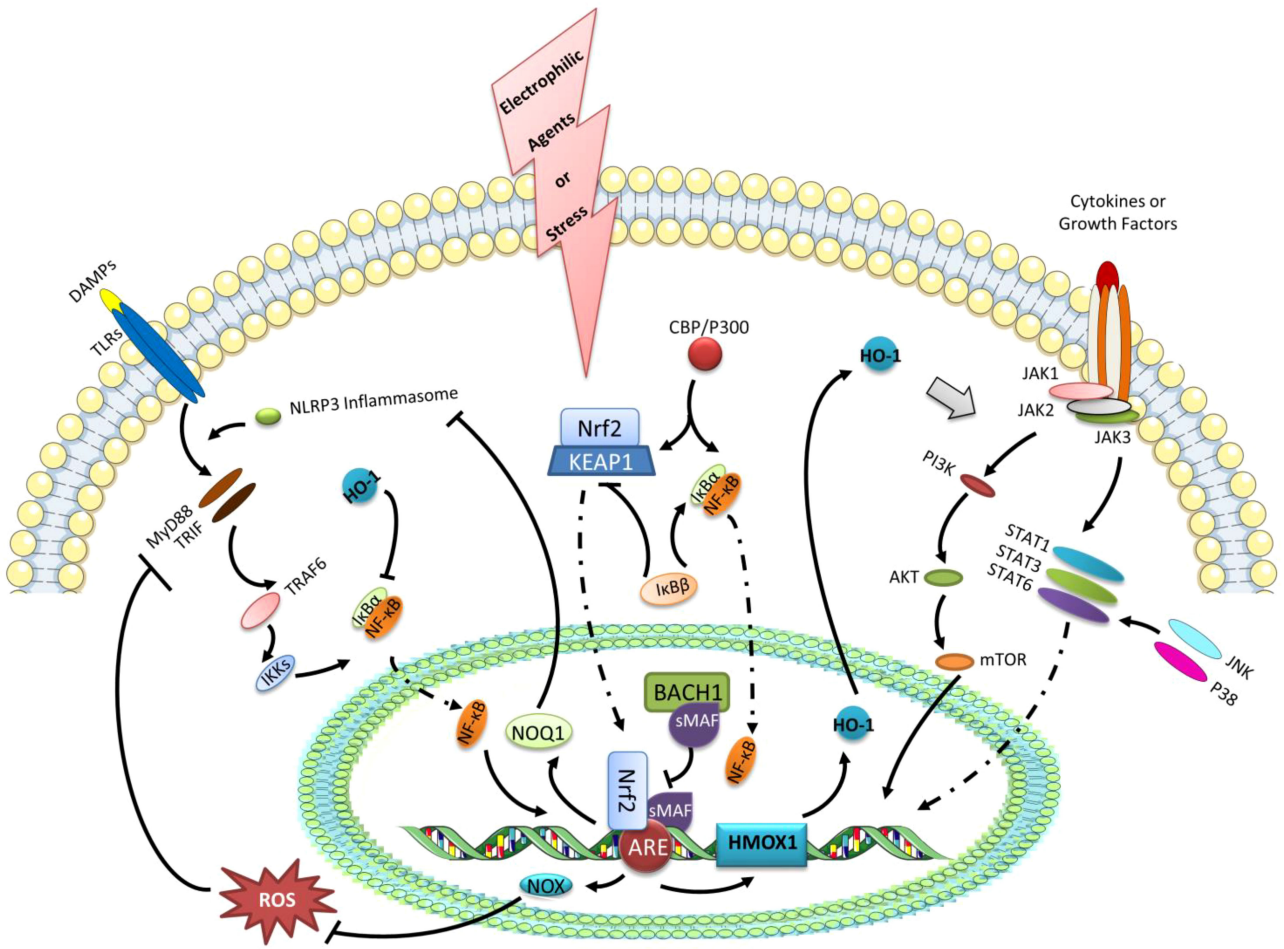
The expression of HO-1 and the negative regulation of NF-κB transcription as the possible pathways of Nrf2 activation controlling macrophage reprogramming. Under the stress or electrophilic agents, Nrf2 can translocate to the nucleus after dissociating from KEAP1. With the accumulation of Nrf2 in the nucleus, it forms a heterodimer with sMAF, which binds ARE, a trans-acting DNA enhancer motif. The Nrf2 binding ARE promotes genes encoding cytoprotective proteins, such as GSH-related enzymes, NAD(P)H dehydrogenase quinone 1 (NQO1), and HO-1. BACH1 is a negative regulator of the inducible HO-1 gene expression, that binds sMAF to inhibit HMOX1 transcription by Nrf2.HO-1 can regulate the polarization of macrophages *via* the STATs signaling pathway. Besides, HO-1 also has negative effects on the transcription of NF-κB. Furthermore, the up-regulated NOX1/2 and NOQ1 by Nrf2 activation can suppress NF-κB transcription *via* eliminating ROS and inhibiting NLRP3 inflammasome, respectively. In addition, IκBβ can stabilize KEAP1, while IκBβ can activate IκBα. Likewise, the competitive or antagonistic relationships also exist in the binding of IκBα and KEAP1 to the common activator CBP/P300.

Furthermore, polarized macrophages can be used to target synovial inflammation caused by OA (5, 20). There is evidence that Nrf2 activation can inhibit M1 macrophage polarization in OA, which indicates that Nrf2 has a protective role in OA synovitis (40). To a large extent, however, the mechanism linking Nrf2-activation in macrophages remains unknown in the condition of OA. In addition to the existing macrophage polarization mechanism and the treatment strategy of OA, several possible pathways of Nrf2 activation controlling macrophage reprogramming will be discussed (**Figure 3**).

### 4.1 Nrf2/HO-1 signaling pathway

Heme oxygenase (HO) is a microsomal enzyme that degrades heme to carbon monoxide (CO), iron, and biliverdin, which plays a protective role in intracellular detoxification during tissue injury. HO regulates a range of anti-inflammatory, antioxidant, and anti-apoptotic pathways through heme degradation, and HO-1 is responsible for most intracellular detoxification among all the HO members (159, 160). The transcription of HO-1 is regulated by the Nrf2/KEAP1/ARE pathway (161), transcription repressor BACH1, AP-1, and several protein kinase signalings (162). Furthermore, HO-1 expression plays a key role in M2 polarization in macrophages (163). For example, activating HO-1 after myocardial infarction can switch M1 macrophages into M2 macrophages (164).

BACH1 plays an important role in Nrf2/HO-1 transcriptional activity (165). BACH1 is a negative regulator of the inducible HO-1 gene expression, that binds sMAF to inhibit HMOX1 transcription by Nrf2, while losing its function in high concentrations of heme (166). BACH1 and Nrf2 competed with each other to regulate ARE-mediated gene expression (167). BACH1-deficient peritoneal macrophages express HO-1 and Arginase-1, Fizz-1, Ym1, and MRC1, which are M2 macrophage markers (168). Nrf2/KEAP1-BACH1 equilibrium has been identified in pulmonary emphysema patients, whereby high levels of BACH1 and KEAP1 result in reduced stress response, mediated by MAPKs, including JNK and ERK (169, 170). The evidence indicates that HO-1 expression mediated by Nrf2 can be regulated strictly by KACH1 in macrophages, and several signaling cascades can control the balance between both, for example MAPKs (171).

The promoter regions of HO-1 genes contain many AP-1 functional sites (163). AP-1 is made up of heterodimers of members of c-Fos and c-Jun (172). Classical AP-1 is located in the consensus sequence of ARE, and the region of ARE is also the main site for Nrf2 to interact with the HMOX1 promoter (173). A growing body of evidence suggests that inhibition of activated AP-1 helps induce HO-1 expression through Nrf2 (174, 175), which is induced by inhibiting c-Fos (176). It is not clear exactly what role AP-1 plays in Nrf-2 activity, but after all, AP-1’s interaction with c-Jun can activate the transcription of GSH-related enzymes and NQO1 (177).

Anti-inflammatory responses in monocytes/macrophages are mediated by an increase in HO-1. Activation of STAT3 and p38/PI3K signaling is necessary to induce HO-1 expression by LPS and IL-10 in rodents (178). LPS-induced activation of HO-1 in M1 phenotypes induces the production of IL-10, as well as the down-regulation of COX-2, iNOS, TNF-α, and IL-6 (179, 180). Inhibition of M1 phenotype with down-regulation of TNF-α induced by globular adiponectin is dependent on IL-10/STAT3/HO-1 pathway in Kupffer cells (181). However, the increase in IL-10 and the decrease in HO-1 have been observed in macrophages stimulated by LPS in humans (182). M1 polarization can be inhibited by HO-1, although this dichotomy suggests that HO-1 controls IL-10 expression in a complex way. Additionally, HO-1 is known to promote M2 phenotype *via* anti-inflammation response and cell protection. HO-1 expression induced by sphingosine-1-phosphate in the supernatant derived from apoptotic cells has been indicated to play a vital role in M2 polarization of macrophages, *via* the STAT1/STAT3 heterodimer (183). Full-length adiponectin induces an M2 phenotype *via* IL-4/STAT6/HO-1, with a decrease in macrophage sensitivity to stimulation by TLR4 ligands, and an increase in M2 markers (184).

### 4.2 Nrf2 and NF-κB signaling

Transcription factor NF-κB has a central role in inflammation, responsible for the promotion of pro-inflammatory mediators and cytokines (281). Nrf2 and NF-κB signaling regulate the redox homeostasis and inflammation responses, that is, the activation of Nrf2 has been identified to functionally couple the inhibition of NF-κB transcriptional activity (29, 185). For example, the up-regulated HO-1 is also a mediator induced by Nrf2 activation with negative regulatory effects on NF-κB (186). On the one hand, HO-1 can inhibit the degeneration of IκB-α leading to the stabilization of NF-κB in cytoplasm (187). On the other hand, CO induced by HO-1 can inhibit the TLR/NF-κB signaling *via* binding downstream transcription factor IRF3 and interfering in the downstream pathway (188). Besides, NF-κB family consists of two types, among which p65 has RelA, RelB, and c-Rel transactivation domains, while p50 and p52 do not have transactivation domains. And for transcription, P50 and P52 need to form heterodimerization with the Rel proteins (189). Notably, novel treatments for inflammatory diseases can inhibit NF-κB and activate Nrf2 to alleviate inflammation (190, 191).

The highly electronegative oxygen can accept electrons generated by normal oxidative metabolism within cells, and ROS are produced, which include superoxide anion (O2 -.), hydrogen peroxide (H_2_O_2_), hydroxyl radical, and singlet oxygen. ROS generated in cytoplasm and mitochondria act their key roles as signaling molecules to regulate physiological processes of macrophages. The ROS generated in cytoplasm and mitochondria serve as important signaling molecules to regulate macrophage physiological processes. NADPH oxidases (NOXs) transfer one electron from NADPH to oxygen to produce cytosolic ROS. Besides, the production of mitochondrial ROS is higher under the biochemical activities of ETC, monoamine oxidases, and P66shc (192). Macrophages could undergo pro-inflammatory cycles promoted by ROS. The targets of ROS include ROS/p38/NF-κB signaling (193), and ROS/p38/STAT1 axis (194), leading to M1 characteristics. And ROS can exacerbate inflammatory response related to NF-κB (p65) (195). However, the role of ROS has two sides. After all, it has been reported that ROS plays a critical role in M2 polarization, during which process the ROS produced by NOX1/NOX2 could contribute to monocyte-to-macrophage differentiation *via* activating of ERK and JNK (196).

Additionally, the activation of Nrf2 can regulate the antioxidant response by controlling the expression of detoxifying enzymes to buffer ROS. Thereby, the decreased ROS results in the inhibition of the activation of NF-κB mediated by oxidative stress (193, 197). Additionally, removing ROS also inhibits STAT1 phosphorylation and conversely activates STAT6 (198, 199). By reducing ROS levels within cells, Nrf2 inhibits oxidative stress-mediated activation, leading to an anti-inflammatory M2 phenotype. Additionally, given the NLRP3 inflammasome signaling as another NF-κB activator, Nrf2 can negatively regulate NLRP3 inflammasome activation (200, 201), especially in the regulative process of ROS. Similarly, Nrf2 can increase NQO1 production and lead to negative effects on NLRP3 inflammasome activation (200). In addition, Hippo-yes-associated protein signaling can also be viewed as a mechanism of Nrf2 activation alleviating inflammation related to NLRP3 inflammasome activation (202).

The mechanisms at a molecular level underlying Nrf2 inhibiting NF-κB are complex. On the one hand, IκB-α is the main inhibitor of NF-κB, which can be phosphorylated by IkB kinase (IKK) β resulting in the release of NF-κB in the pro-inflammation micro-environment. IKKβ can bind KEAP1 *via* the ETGE motif to mediate the ubiquitin and proteasome degradation. KEAP1 is the main inhibitor of Nrf2. When IKKβ is stabilized, KEAP1 can be inhibited, while IκB-α can be phosphorylated, which results in the inhibition of Nrf2 and the activation of NF-κB (203–205). On the other hand, the p65 subunit of NF-κB has been indicated as a partner of KEAP1, and the interaction between the both can inhibit Nrf2-ARE pathway (206). NF-κB (p65) also exerts negative effects on Nrf2 signal transduction by competing for binding with CBP/P300 between p65 and Nrf2. CBP/p300 is not only a co-activator of NF-κB and Nrf2, but also can negatively regulate the biological activity of ARE in the process of transcription under the action of p65 (207, 208). Conversely, the interaction between p65 and KEAP1, and the role of CBP have also been reported as the mechanisms by which Nrf2 negatively regulates NF-κB (209, 210). As a result, the Nrf2 activation can inhibit the production of pro-inflammatory cytokines induced by NF-κB (39, 211). The activated Nrf2 can decrease the release of IL-6 and IL-1β from macrophages by blocking pro-inflammatory cytokine transcription in macrophages (211). Furthermore, M2 polarization induced by the activation of Nrf2 has been reported to treat OA (212).

### 4.3 Potential treatment strategies for OA linking Nrf2 activation

#### 4.3.1 Itaconate and fumarate derivatives

Fumarate is a mitochondrial metabolite acting as the terminal electron acceptor in the ETC of mammalians (213). In the Krebs cycle, succinate dehydrogenase (SDH) catalyzes the oxidation of succinate to fumarate (214). SDH-induced succinate oxidation can switch the production of ROS and the activation of M1 macrophages (215). And then, the isocitrate dehydrogenase in the Krebs cycle is blocked, inhibiting the canalization of cis-aconitate to isocitrate (216). The accumulated cis-aconitate binding to immune-responsive gene 1 can generate itaconate *via* decarboxylation (214, 217). Itaconate is an endogenous metabolite produced during the TCA cycle, which activates Nrf2 *via* the alkylation of KEAP1 to influence macrophage function (218).

4-octyl itaconate (OI) is an esterified itaconate derivative that can be converted into itaconate by a direct modification of intracellular cysteines (219, 220). Dimethyl fumarate (DMF) is an esterified fumarate derivative, which can be rapidly metabolized into monomethylfumarate (MMF) *in vivo* (220, 221). In macrophages, OI and DMF inhibit pro-IL-1β and NLRP3 signaling pathways activated by TLR4 binding DAMPs (222). However, different from OI, the promoting effect of DMF on KEAP1 alkylation and Nrf2 nuclear accumulation comes from its metabolite MMF (223).

OI and DMF have protective effects on the cartilage in OA. More specifically, OI-induced transcription of Nrf2 in chondrocytes results in the high expression of HO-1, NQO1, and GCLC, and the low secretion of IL-6, IL-10, MCP-1, and TNF-α, which can switch the prevention from cell death and apoptosis of chondrocytes *via* decreasing oxidative stress and inflammation responses, and put a brake on the progress of OA *in vivo* (224, 225). Similarly, dimethyl fumarate (DMF) can suppress the production of MMP-1, MMP-3, and MMP-13 and the destruction of COL2 induced by TNF-α in OA, which appears to work by inhibiting JAK/STAT3 signaling (226). Moreover, recent evidence shows that exogenous itaconate promotes polarization of M2 macrophages and reduces apoptosis in chondrocytes, a process in which Nrf2 activation-induced stimulator of interferon genes (STING) suppresses transcription of NF-κB (212).

#### 4.3.2 Curcumin

Curcumin is a yellow-colored lipid-soluble polyphenol that is the main active ingredient extracted from the rhizome of Curcuma longa (also known as turmeric) (227). Curcumin has been shown to reduce inflammation and alleviate oxidative stress, where Nrf2 plays a vital role (228). By increasing Nrf2 transcription and HO-1 synthesis, curcumin protects cells from ROS-induced damage and reduces COX-2 production (229, 230). Of note, macrophage COX-2 is an inflammatory enzyme catalyzing the formation of prostaglandins and thromboxane, which is upregulated during pro-inflammatory conditions (231).

The effects of curcumin in OA can also be independent of the Nrf2/HO-1 axis in reducing inflammation, preventing ECM degradation, and promoting cartilage synthesis (232, 233). The effects on inflammation can be induced by suppressing the expression of pro-inflammatory mediators, such as TNF-α, IL-1β, IL-6, IL-17and TGF-β (233–236), which are mainly regulated by NF-κB signaling. Besides, the degeneration of IκBα and the expression of COX-2 in macrophages are reduced by curcumin, resulting in the reduced transcription of NF-κB lower responses to LPS and M1 polarization (236), implying the activation of Nrf2. Curcumin can upregulate the expression of COL2 and downregulate MMP1, MMP3, and MMP13 in ECM protection (235, 237). In addition, the higher level of mRNA related to cartilage anabolism, including COL2A1 and ACAN, can characterize the effect of curcumin on cartilage regeneration (232).

#### 4.3.3 Quercetin

Similar to curcumin, quercetin is one of the most studied and abundant flavonoids found in tea, vegetables, and fruits (238). Quercetin has a high antioxidant activity due to its capacity to up-regulate the transcription of Nrf2, *via* promoting the degeneration of KEAP1 (239). Quercetin can induce the conversion of macrophages from M1 to M2, characterized by lower levels of iNOS-positive cells and inflammatory mediators, and higher levels of CD206-positive cells in diabetic wound healing (240). By scavenging ROS, quercetin-loaded ceria nanocomposite can also increase the M2/M1 ratio of macrophage polarization in periodontal inflammation models (241). Besides, quercetin has been suggested to suppress the expression of iNOS and COX-2, as well as the secretion of IL-6, TNF-α, and IL-1β of M1 macrophages with the decrease in ROS and chemokines related to M1 polarization, including CCL2 and CXCL10, while IL-10 of M2 macrophages has been found up-regulated, with the increase in HO-1 and NQO1 *via* AMPK and AKT signaling pathways (242). Notably, potential mechanisms underlying the effects of quercetin in the immunoregulation of macrophages can also involve the inhibitive effects on the activity of NLRP3 inflammasome *via* TLR2/Myd88/NF-κB and ROS/AMPK pathway (243).

In OA, the effects of quercetin on anti-inflammation response and cartilage protection have been addressed (244). Quercetin can act its anti-inflammatory role in suppressing NO, TNF-α, and IL-1β through inhibiting the NLRP3 signaling pathway, p38 activation, and endoplasmic reticulum stress (245–247). In addition, the activated SIRT1/AMPK signaling pathway can not only suppress the apoptosis of chondrocytes related to endoplasmic reticulum stress, but can also mediate the reversion of mitochondrial dysfunctions and the elimination of ROS in chondrocytes (246, 248). Along aside with anti-inflammation response, the inhibition of cartilage degeneration and the promotion of cartilage regeneration can be characterized by the down-regulation of MMP3, MMP9, MMP13, and ADAMTS-5, while the up-regulation of COL2 (247, 249). Of note, it has been implied that the effects of quercetin can be better loaded by Nano-materials (249, 250). Furthermore, the synovial level of TGF-β1 and TGF-β2 has been found up-regulated after the administration of quercetin due to the increase in M2 macrophages, which can also promote the production of IGF and build a microenvironment promoting chondrogenesis (248).

#### 4.3.4 Melatonin

N-acetyl-5-methoxytryptamine, also known as Melatonin (Mel), is an indolamine with numerous functions in neural, endocrine, and immune physiological activities, playing a versatile role in the regulation of circadian rhythms, the defense against oxidative and inflammation, and the modulation of mitochondrial homeostasis. Mel is synthesized from tryptophan under 5-hydroxytryptamine in multiple extrapineal tissues (251, 252). In OA, Mel has been considered a novel treatment for OA due to its effects on the protection of chondrocytes from apoptosis, the promotion of anabolic metabolism, and the suppression of catabolic metabolism in cartilage, the restoration of redox balance, and the regulation of sirtuin signaling pathways (253).

The role of Mel in the immunoregulation of macrophages has not been verified, however, several studies support this idea. In macrophages, the administration of Mel can induce the activation of Nrf2 and the increased expression of HO-1, while the expression of iNOS and COX-2, and the production of TNF-α, IL-1β and IL-6 can be reduced through the inhibition of NF-κB transcription (254, 255). Besides, the reduced activation of STAT1 and the increased STAT3 can drive the transformation from M1 to M2 phenotype (256). Five potential mechanisms underlying shaping polarization of macrophages have been implied by the review, including through cellular pathways of JAK/STAT, cellular metabolism, miRNAs, mitochondrial dynamics, and mitophagy (257). Furthermore, the most recent study indicates that the interaction of Mel and MT_1_ receptors can activate PI3K/Akt and ERK signaling pathways in synovial fibroblasts leading to the up-regulation of microRNA-185a, which can reverse OA-induced pathology in animal models through reducing the secretion of TNF-α, IL-8, and vascular endothelial growth factors (258).

#### 4.3.5 Mesenchymal stem cells

Mesenchymal stem cells (MSCs) are heterogeneous stromal cells commonly sourced from adipose tissue, bone marrow and umbilical cord blood. MSCs have the capacity of differentiating into adipocytes, chondrocytes, and osteoblasts. Except for the capacity of multidirectional differentiation, MSCs exert broad immunoregulatory abilities, which can induce the specific polarization (M1 to M2) of macrophages to promote the repair of damaged tissues *via* cell-to-cell contact and paracrine actions (259–262). The mechanisms and evidence of MSCs mediating the alterations of macrophage phenotypes have been reviewed. Furthermore, the Hippo pathway activating Yes-associated proteins was pointed out as a new mechanism of inhibiting NLRP3 signaling underlying MSCs regulating macrophages (263).

In OA, several studies have revealed the potential effects of MSCs on regulating intra-articular inflammation *via* M2 macrophage polarization. MSCs derived from bone marrow, which are labeled by iron oxide nanoparticles, can induce the increase in CD206-positive cells out of F4/80-positive macrophages, while can also induce the decrease in iNOS-positive cells out of F4/80-positive macrophages in animals experiencing OA due to destabilization of medical meniscus (264). Besides, the cell-free fat extract as a derivative of adipose-derived stem cells, which is rich in cytokines and nutrients, has been reported to be dose-dependently effective in relieving pain (tested by behavioral tests of rats), protecting cartilage, and increasing the ratio of M2 phenotype in the synovium (CD206-positive macrophages) in rat models with sodium iodoacetate-induced OA. The same study also revealed that the cell-free fat can decrease the ratio of CD86-positive cells, and reduce iNOS and COX-2 induced by LPS and IFN-γ in Raw 264.7. And then IL-6 and ADAMTs-5 were reduced, while the expression of SOX-9 was promoted in chondrocytes administrated by the cell-free fat. In addition, ROS can be regulated in these processes (265).

Notably, specifically pre-conditioned MSCs seem to show better regulation of macrophages. MSCs-derived extracellular vesicles (EVs) with antioxidative characteristics *via* over-expressing Nrf2 in adipose MSCs have effects on anti-inflammation and antioxidation. These EVs can induce increased levels of M2 macrophages, and decreased IL-6 and TNF-α (266). According to the International Society for Extracellular Vesicles, EVs are lipid bilayer membrane particles naturally released by the cells. EVs contain proteins, lipids, and nucleic acids, while without a functional nucleus (267). For MSCs, the production of EVs contributes to regulating the activation states of macrophages. The main active substances that play a regulatory role are miRNA and mitochondria transferred through EVs. miRNAs could target various transcription factors (for example, NF-κB) and adaptor proteins (for example, IL-1β) at the post-transcriptional level. Besides, the mitochondrial transfer could be related to the promotion of oxidative phosphorylation and the repair of oxidative stress function (268, 269). The acute lung injury has been ameliorated by MSC-derived small EVs (MSC-EVs) *via* activating Nrf2, during which process the increase in immune and redox mediators, including TLR4, Arg1, and HO-1 could be revealed (270). Besides, in a recent study, nanoparticles simulating EVs are effective for OA by promoting the polarization from M1 to M2. The structure of these EVs is oxidative stress-responsive bilirubin grafted polylysine biomaterial vesicles containing immunoglobulin IgG and berberine (271). However, there is still a knowledge gap in MSC-EVs regulating macrophage polarization *via* Nrf2 in OA treatment. Besides, the expression of Nrf2 in MSCs was promoted by hypoxic preconditioning, while the expression of NF-κB was reduced. And the intrarenal transplantation of these hypoxic preconditioned MSCs was more effective in the activation of HIF-1α/VEGF/Nrf2 signaling to reduce glomerular apoptosis, autophagy, and inflammation (272). However, the evidence of the Nrf2 activation in macrophage has been clearly pointed out in another study. Bone marrow-derived MSCs pre-conditioned by FNDC5, a transmembrane protein acting a crucial role in inflammation diseases, have been found to produce more exosomes. These pre-conditioned exosomes have shown the effects of promoting M2 macrophages and anti-inflammation response in myocardial infarction *via* the inhibition of NF-κB signaling pathway and the activation of Nrf2/HO-1 axis (273).

#### 4.3.6 Low-intensity pulsed ultrasound

Low-intensity pulsed ultrasound (LIPUS) outputs in a pulse wave mode of ultrasound with a non-thermal effect, at an intensity lower than 3 W/cm^2^ (274). The use of LIPUS in OA treatment has been reported as an effective manner in protecting cartilage from degeneration *via* reducing the expression of MMP3, MMP13, and TGF-β1 (275). LIPUS has been considered an effective strategy for patients with OA, which was verified by a recent randomized clinical trial (276).

Besides, LIPUS can promote the cartilage differentiation of bone marrow-derived MSCs due to promoting the nuclear localization of SOX9 dependent on the phosphorylation of ERK1/2 (277). The combination of nanoparticles and LIPUS has been implied to have better effects on OA through inhibiting the degeneration of cartilage (278, 279). Of note, the effects of LIPUS on immunoregulation in macrophages have been reviewed as a potential mechanism of treating OA (20), however, there was little direct evidence. A most recent study has revealed that LIPUS can significantly suppress the secretion of IL-1β, IL-6, and TNF-α induced by LPS in macrophages derived from bone marrow, which can be attributed to increasing the level of intracellular itaconate and the expression of Nrf2 (280).

## 5 Concluding remarks

In this review, we reviewed the role of macrophage phenotypes in driving and relieving inflammation due to OA and summarized how NF-κB, Nrf2, and their crosstalk shape macrophage polarization. As Nrf2 signaling is believed to impact cellular metabolism, studying the effects of activators of Nrf2 on macrophage metabolism and phenotype related to inflammation could reveal how OA can be treated through reprogramming macrophage functions. Furthermore, studying the effect of Nrf2 activation on macrophages in the OA microenvironment may suggest a potential anti-inflammatory therapy target. Therefore, it is imperative to identify the relationship between Nrf2, macrophage function, and the progression of inflammatory diseases.

## Author contributions

Conception and design of study, LW and CH. Drafting of article, LW. Revision of draft, CH. All authors contributed to the article and approved the submitted version.

## Funding

This work was supported by the National Natural Science Foundation of China (81972146 to CH), the Department of Science and Technology of Sichuan Province (2020YJ0210 and 2021YFS0004 to CH).

## Conflict of interest

The authors declare that the research was conducted in the absence of any commercial or financial relationships that could be construed as a potential conflict of interest.

## Publisher’s note

All claims expressed in this article are solely those of the authors and do not necessarily represent those of their affiliated organizations, or those of the publisher, the editors and the reviewers. Any product that may be evaluated in this article, or claim that may be made by its manufacturer, is not guaranteed or endorsed by the publisher.
